# Social support and gender differences in coping with depression among emerging adults: a mixed-methods study

**DOI:** 10.1186/s13034-015-0088-x

**Published:** 2016-01-07

**Authors:** Angel Martínez-Hernáez, Natàlia Carceller-Maicas, Susan M. DiGiacomo, Santiago Ariste

**Affiliations:** Medical Anthropology Research Center, Universitat Rovira i Virgili, Avinguda de Catalunya, 35, 43002 Tarragona, Spain; Department of Anthropology, Philosophy and Social Work, Universitat Rovira i Virgili, Avinguda de Catalunya, 35, 43002 Tarragona, Spain; Department of Anthropology, Machmer Hall, University of Massachusetts at Amherst, Amherst, MA 01003 USA; Department of Psychology, Universitat Rovira i Virgili, Avinguda de Catalunya, 35 43002 Tarragona, Spain

**Keywords:** Emerging adulthood, Depression, Social support, Emotional distress, Mixed-methods study

## Abstract

**Background:**

Depression affects a considerable proportion (12–25 %) of adolescents and so-called emerging adults (ages of 18 and 25). The aims of this study were to explore the relationship between perceived social support and depression in a sample of emerging adults, and subsequently to identify the type of social support young people consider most helpful in dealing with this type of emotional distress.

**Methods:**

A sample of 105 young persons (17–21 years of age) was selected from a previous longitudinal study to create three groups of participants: subjects with a previous diagnosis of depression; subjects with self-perceived but undiagnosed distress compatible with depression; and a group of controls. Qualitative and validated instruments for measuring depressive symptoms (the BDI-II, Beck depression inventory) and social support (the Mannheim interview on social support) were administered.

**Results:**

Loss of friendships over time and dissatisfaction with social and psychological support are variables associated with depression in emerging adulthood. Qualitative analysis revealed gender differences both in strategies for managing distress, and in how social support was understood to mitigate depressive symptoms. Male study participants prioritized support that helped them achieve self-control as a first step toward awareness of their emotional distress, while female study participants prioritized support that helped them achieve awareness of the problem as a first step toward self-control.

**Conclusions:**

Treatment for emerging adults with depression should take into account not only the impact of social support, but also gender differences in what they consider to be the most appropriate form of social support for dealing with emotional distress.

**Electronic supplementary material:**

The online version of this article (doi:10.1186/s13034-015-0088-x) contains supplementary material, which is available to authorized users.

## Background

Depression affects a considerable proportion (12–25 %) of adolescents and so-called emerging adults (ages of 18 and 25) [1], and has clinical and psychosocial implications that include a higher risk of suicide, substance abuse, social adjustment problems, reduced academic performance, lower career satisfaction, and a greater risk of severe mental disorder in adult life [2, 3]. It is estimated that the lifetime prevalence of depression and dysthymia increases by 15.4 % in young people between the ages of 17 and 18 years, and that the incidence and cumulative prevalence of these problems among emerging adults reaches 25 % [4–6]. Nevertheless, adolescents and emerging adults constitute the age groups least likely to avail themselves of professional mental health care services for treatment of their depressive symptoms, and those that place the greatest trust in their social networks to resolve them [7–10].

Social relations, variously categorized as social ties, social networks, social support or social capital, constitute one of the most important and frequently studied social determinants of health and mental health [11, 12]. Social support is understood as the help provided by individuals who comprise the social network of a person who occupies the position of ego in this network. A distinction is made between perceived and received support, as well as between psychological/emotional support on the one hand and instrumental support on the other. Social support, therefore, is the functional dimension of the social network, which is not limited to a collection of egocentric ties that vary in the number, intensity and frequency of contacts, but may be broadened to include the wider context of the community as a network of networks, and to social capital, understood as the possible benefits both for individuals and for groups resulting from mutual cooperation and collaboration.

The many studies of social support demonstrate the relevance of social ties in the onset, course and mitigation of depressive symptoms in diverse age groups and social contexts [13]. Social support has a positive effect on the clinical course of depression [14], facilitating recovery from major depression [15], and its absence is a predictor of a greater incidence of depressive symptoms in the general population and of a worse prognosis in diagnosed patients [16]. In addition, it is known that neighborhood social ties affect depression outcomes [17], whether through the formation of protective support networks that favor agency and self-control or by encouraging trust, which has a positive and protective impact on friendships.

Among adolescents and emerging adults, peer and parental support is inversely associated with factors such as the risk of suicide attempts among depressed outpatients [18] and the onset of depressive symptoms [19]. It has also been observed that social support moderates the impact of stress on depressive symptoms [20]. The role of social support, however, remains in many ways unspecified. For example, women (including adolescents and young adults) have more close social ties than men, mobilize more social support in situations of stress and crisis, and offer more support than men in these situations [21, 22]; in comparison to men, however, they have a higher incidence and prevalence of depression at any age.

Additionally, most studies of social support are carried out with standardized instruments for measuring perceived social support that neither include structural variables such as the size and density of social networks or the frequency of social contacts, nor incorporate the views of the social actors on what forms of social support they consider most essential for resolving their distress.

This study explores, in a sample of emerging adults, different dimensions of the relationship between social support and depressive symptoms, with two objectives. The first objective is to analyze the association between perceived social support, social networks and depressive symptoms using quantitative techniques. The second objective is to learn what type of social support emerging adults consider most helpful in resolving depressive emotional distress, a question we address through qualitative methods.

## Methods

### Research design and sample selection

The emerging adults in this exploratory study were recruited from the Panel de Famílies i Infància (PFI), a four-wave longitudinal sociological study designed by the Consorci Institut d’Infància i Món Urbà (CIIMU) [23]. It was initiated in 2006 with a representative sample of 3004 adolescents born between 1990 and 1993 and resident in Catalonia, and incorporated a new cohort every year. The present study may be considered the fifth wave. Information was collected on negative mood states using a self-administered scale (years 2007 and 2008), the presence or absence of a diagnosis of depression (years 2006 and 2010), and patterns of sociability and economic, school, and family factors (during all four waves).

For this study, a sub-sample of the PFI was recruited from all over Catalonia, rural areas included, using the propensity matching score technique in order to yield three groups of 50 participants each: one with depression diagnosed by a health professional in the first or fourth wave of the PFI, as reported by the parents in response to a direct question; a second group with self-perceived depressive distress (feeling sad, lonely and “down” on a frequent basis) in the second and third wave but without a diagnosis of depression; and a control group with neither self-perceived distress nor a psychiatric diagnosis. In order to select the sample, three segments were created (individuals with a diagnosis, individuals with self-perceived distress, and controls) from the database using homogeneous criteria for gender, age, and socioeconomic status of the domestic group, and 50 subjects were chosen from each of the three segments via simple random sample without replacement. Sample attrition occurred in cases of change of residence, inability to contact the subject, or subjects who declined to be interviewed, and in the end 105 subjects were interviewed: 37 with a diagnosis, 33 with self-perceived distress, and 35 controls. The gender (Chi square: 2.041; p value: 0.153) and age (Chi square: 2.613 p value: 0.455) characteristics of the missing subjects were not significantly different from those of the subjects interviewed. We considered the possibility of recruiting more participants if the data saturation point was not reached in qualitative analysis, but this proved unnecessary.

The study procedures were approved by the ethics committee of the Fundació Congrés Català de Salut Mental, an interdisciplinary entity for the promotion of mental health, and carried out in accordance with the ethical standards established by the Helsinki Declaration. Each participant and one adult with parental responsibility provided written informed consent.

### Instruments

In this study various different instruments were used to analyze social networks, social support and the existence, either past or present, of symptoms of depression and emotional distress.

#### The sociological questionnaires

For this study, we analyzed the variables of sociability and previous experiences of depression and emotional distress obtained from the sociological questionnaires used in the previous four waves. Specifically, we included the presence (1) or absence (0) of a diagnosis of depression in waves 1 and 4 of the PFI, as well as the presence or absence of emotional distress in waves 2 and 3. We also included various sociability variables such as the number of friendships in the different waves.

#### The Beck Depression Inventory (BDI-II)

Symptoms of depression were assessed using the Beck Depression Inventory (BDI-II), an instrument that has been widely used as a measure both in patients with mental disorders and in the general population [24, 25]. According to the manual of the BDI-II, scores from 0–13 indicate minimal depression, scores from 14–19 indicate mild depression, scores from 20–28 indicate moderate depression, and scores from 29–63 indicate severe depression. In this study we used the version validated for Spanish-speaking contexts. The data were dichotomized into two broad categories: moderate/severe depression (1) versus mild/minimal depression (0), a decision justified by the fact that in some studies the optimal cut-off score for differentiating between individuals with and without depressive disorder is in the range of ≥21 [26, 27].

#### The Mannheim Interview on Social Support

Social support was assessed with the Mannheim Interview on Social Support (MISS), a structured interview that addresses both structural (social network) and functional (social support) dimensions [28]. It has been validated for Spanish-speaking contexts and is highly reliable [29]. The variables utilized in this study were: psychological everyday support (PES); instrumental everyday support (IES); psychological crisis support (PCS); and instrumental crisis support (ICS). In addition, we included structural measures of social networks: number of friendships, and conflictive relationships with friends and family members.

#### The qualitative questionnaire

We used a qualitative semi-structured questionnaire (see Additional file 1: Appendix S1) in order to explore the strategies used by young people to deal with depressive types of distress, including the type of social support they considered most helpful, and other factors such as lay explanatory models of depression and preferred help-seeking processes. The items included in the questionnaire were agreed upon by the research team with the advice of several mental health professionals in the course of three joint meetings. The questions were formulated in accordance with the aims of the study and by consensus among the members of the research team and the mental health professionals following a thoroughgoing review of the available literature.

#### Focus groups

Three focus groups were organized, each comprising four to eight previously interviewed young adults of both sexes representing all three subgroups (diagnosis, undiagnosed distress, and control). At each session the preliminary results of the interviews were presented in order to facilitate a comparative discussion of the data obtained from the qualitative questionnaire. Additionally, we organized two focus groups of professionals and one mixed group including both young people and professionals with the purpose of creating a guidebook of best practices and a documentary video [30].

#### Interviewers

The 11 interviewers, all of whom were researchers in medical anthropology and/or psychology, participated in two working sessions to unify criteria and coordinate the dynamics of fieldwork and interviews. The interviews were carried out in Spanish or in Catalan, depending on the subject’s mother tongue. Interviewing was carried out between March and October 2011 at the convenience of the participants, who were contacted by telephone. Each interviewer wrote up a reflexive evaluation of every interview completed. The interviewers were trained by the research team in order to ensure reliability in the administration of both the psychological scales and the qualitative questionnaire. The psychological scales were evaluated and analyzed by the psychologists participating in the project.

The focus groups took place between April and June 2012 in a room prepared for this purpose in a civic center in Barcelona. Each group included a moderator and a note-taker, in both cases persons with training and experience in facilitating focus groups and in the ethnographic approach. The focus groups were useful for corroborating the results of the preliminary analysis of the qualitative questionnaire.

### Analysis

The quantitative data were analyzed using SPSS Statistics Version 20 software. The information was codified and introduced into a data base that was later combined with the original data base of the PFI, which contained 2416 variables derived from previous interviews with the 105 study participants. In the first phase, consistency analysis was used to create new variables to retrieve information not only from the present wave but from earlier waves as well. Because of the small size of the sample and the exploratory nature of the study, the ORs were calculated by bivariate analysis, for all variables. The same analysis was applied to the sample following segmentation by gender group. In order to avoid measurement errors we controlled the effect of outliers in the sample.

The qualitative data were managed using ATLAS.ti 6.2.27 software [31]. Through group discussion of our observations, we did an initial thematic analysis to identify the main themes present in the data. We then established a structure for coding in accordance with the principles of grounded theory and the ethnographic method, including the identification of native or emic typologies. We reviewed the interview transcripts and applied the codes. Several methods were used to enhance the rigor of our analyses, including identification and analysis of the exceptions, the constant comparative method, accrual of subjects beyond theme saturation, and the principle of reflexivity. The results obtained from the qualitative questionnaire were compared with those obtained from the focus groups.

## Results

Table 1 shows the characteristics of the study participants. The emerging adults in our study were between 17 and 21 years of age and the majority were female (68.6 %). All cases (n = 5) of severe depression according to the BDI-II were located in the group with a previous diagnosis of depression. The participants with previous distress had more limited social networks, both of family members and of peers, as can be observed in this table.

**Table 1:** Sample studied

	Participants with diagnosis		Participants with mental distress		Participants with mental distress		Total		Statistics		
	n	%	n	%	n	%	n	%	Chi square distribution	gl	Sig.
Male	13	35.1 %	9	27.30 %	11	31.4 %	33	31.4 %	0.5	2	0.779
Female	24	64.9 %	24	72.70 %	24	68.6 %	72	68.6 %			
17 years	5	13.5 %	3	9.10 %	7	20.0 %	15	14.3 %	Chi square distribution	gl	Sig.
18 years	10	27.0 %	6	18.20 %	5	14.3 %	21	20.0 %	6.571	8	0.584
19 years	12	32.4 %	10	30.30 %	9	25.7 %	31	29.5 %			
20 years	7	18.9 %	9	27.30 %	12	34.3 %	28	26.7 %			
21 years or older	3	8.1 %	5	15.20 %	2	5.7 %	10	9.5 %			
Under 18,000 €	6	16.2 %	5	15.60 %	8	23.5 %	19	18.4 %	Chi square distribution	gl	Sig.
18,001–36,000 €	18	48.6 %	13	40.60 %	13	38.2 %	44	42.7 %	3.915	6	0.688
36,001 € or more	9	24.3 %	7	21.90 %	5	14.7 %	21	20.4 %			
Missing data	4	10.8 %	7	21.90 %	8	23.5 %	19	18.4 %			
Single-parent family	11	29.7 %	7	21.20 %	14	40.0 %	32	30.5 %	Chi square distribution	gl	Sig.
Two-parent or reconstituted family	17	45.9 %	19	57.60 %	19	54.3 %	55	52.4 %	6.563	4	0.161
No information	9	24.3 %	7	21.20 %	2	5.7 %	18	17.1 %			
Minimal	20	54.1 %	24	72.70 %	30	85.7 %	74	70.5 %	Chi square distribution	gl	Sig.
Mild	8	21.6 %	4	12.10 %	3	8.6 %	15	14.3 %	15.534	6	0.016
Moderate	4	10.8 %	5	15.20 %	2	5.7 %	11	10.5 %			
Severe	5	13.5 %	0	0.00 %	0	0.0 %	5	4.8 %			
Average	19.30		19.67		19.63		19.52		F	gl	Sig.
N	37		33		35		105		1.286	2	0.281
Standard deviation	0.87765		1.16369		1.16533		1.07502				
Average	8.79		8.15		8.76		8.57		F	gl	Sig.
N	34		33		34		101		2.132	2	0.124
Standard deviation	5.432		4.515		3.782		4.59				
Average	5.15		4.24		4.88		4.76		F	gl	Sig.
N	34		33		34		101		0.205	2	0.815
Standard deviation	2.105		1.64		1.737		1.861				

The results presented in Table 2 show that no statistically significant association was found between a previous diagnosis of depression and moderate/severe depression according to BDI-II scores at the time of the study at p < 0.05, but was present at p < 0.10. While some mental disorders may become chronic, they may also fluctuate in relation to different life circumstances, or have forms of clinical expression not captured at the time of the interview. Our data do, however, show a statistically significant association between a BDI-II score higher than minimal and a previous diagnosis of depression (OR: 3.28 CI 95 %: 1.37–7.86, p < 0.01), an association that is maintained when we group the study participants who had a previous diagnosis together with those who had depressive emotional distress (OR: 3.55, CI 95 %: 1.22–10.27, p < 0.01). Similarly, among the female study participants there was a robust association between moderate/severe depression according to BDI-II scores and a previous diagnosis of depression (OR: 8.543, CI 95 %: 1.045–69.82, p < 0.05).

**Table 2:** Bivariate analysis

	1	2	3	4	5	6	7	8	9	10
1. Diagnosis vs. self-perceived distress and controls										
2. Severe to moderate BDI-II score vs. mild to minimal BDI-II score	Odds: 2.8 CI (0.95–8.28)+									
3. Fewer friends at 5th than at 1st wave vs. the same or more friends	Odds: 1.17 CI (0.51–2.69)	Odds: 8.47 CI (1.8–39)**								
4. Eight or fewer friends at MISS vs. more than eight friends	Odds: 0.81 CI (0.35–1.85)	Odds: 2.67 CI (0.8–8.93)	Odds: 2.89 CI (1.27–6.5)*							
5. Dissastified with psychological everyday support (PES)	Odds: 2.77 CI (1.14–6.73)*	Odds: 5.46 CI (1.1–25)*	Odds: 1.6 CI (0.71–3.61)	Odds: 0.85 CI (0.38–1.9)						
6. Dissatisfied with instrumental everyday support (IES)	Odds: 1.24 CI (0.52–2.95)	Odds: 1.1 CI (0.35–3.48)	Odds: 1.09 CI (0.46–2.62)	Odds: 1.57 CI (0.64–3.86)	Odds: 1.86 CI (0.76–4.59)					
7. Dissatisfied with instrumental crisis support (ICS)	Odds: 0.93 CI (0.42–2.08)	Odds: 1.97 CI (0.63–6.12)	Odds: 0.9 CI (0.41–1.99)	Odds: 0.7 CI (0.32–1.56)	Odds: 1.54 CI (0.7–3.38)	Odds: 1.18 CI (0.5–2.75)				
8. Dissatisfied with psychological crisis support (PCS)	Odds: 2.79 CI (1.12–6.94)*	Odds: 4.51 CI (1.4–14)**	Odds: 3.2 CI (1.19–8.56)*	Odds: 3.25 CI (1.1–9.05)*	Odds: 3.6 CI (1.2–10.5)*	Odds: 2.66 CI (1.05–6.74)*	Odds: 4.65 CI (1.59–13.59)**			
9. Conflict with family	Odds: 1.37 CI (0.61–3.06)	Odds: 1.91 CI (0.64–5.71)	Odds: 1.23 CI (0.56–2.71)	Odds: 1.19 CI (0.54–2.63)	Odds: 1.66 CI (0.75–3.66)	Odds: 0.3 CI (0.12–0.7)**	Odds: 0.86 CI (0.4–0.87)	Odds: 1.33 CI (0.55–3.24)		
10. Conflict with peers	Odds: 1.11 CI (0.47–2.62)	Odds: 1.66 CI (0.49–5.59)	Odds: 0.72 CI (0.31–1.66)	Odds: 0.24 CI (0.9–0.6)**	Odds: 2.48 CI (1.08–5.73)*	Odds: 0.91 CI (0.38–2.2)	Odds: 1.2 CI (0.53–2.71)	Odds: 1.15 CI (0.44–3)	Odds: 0.84 CI (0.3–1.9)	

CI (confidence interval 95 %), ** p < 0.01, * p < 0.05, + p < 0.10

### Depression and social support

Bivariate analysis (Table 2) shows that both participants with a previous diagnosis of depression and those with severe or moderate depression according to BDI-II were less satisfied with PES and PCS, but not with instrumental support, and this was the case for both IES and ICS. Loss of friendships, understood as having fewer friends in the fifth wave than in the first, was associated with a severe to moderate BDI-II score (OR: 8.47, CI 95 %: 1.81–39.63, p < 0.01), especially among the participants with a previous diagnosis (OR: 10.400, CI 95 %: 2.033–53.202, p < 0.01) and among the female study participants (OR: 16.792 CI 95 %: 2.051–37.481, p < 0.01).

For the study participants in general, there was no statistically significant association between the number of friendships according to the MISS and the depression variables (a previous diagnosis and moderate/severe depression according to the BDI-II). There was no statistically significant association between these variables and the existence of conflictive relations with family members and friends.

Table 3 shows that the symptoms of depression associated with lack of satisfaction with PES were sadness, self-criticalness, crying, and changes in sleep pattern, and those linked to lack of satisfaction with PCS were pessimism, guilt feelings, punishment feelings, crying, agitation, and feelings of worthlessness. Loss of friendships between waves 1 and 5 was associated with feelings of sadness and self-criticalness. When these associations were analyzed separately by gender, we observed that:

**Table 3:** Social network/social support variables and symptoms of depression

	Fewer friends in the fifth than in the first wave				Less psychological everyday support (PES)				Less psychological crisis support (PCS)			
	Value (ODDS)	Confidence interval 95 %		Sig. asymptotic (bilateral)	Value (ODDS)	Confidence interval 95 %		Sig. Asymptotic (bilateral)	Value (ODDS)	Confidence interval 95 %		Sig. Asymptotic (bilateral)
		Lower boundaries	Upper boundaries			Lower boundaries	Upper boundaries			Lower boundaries	Upper boundaries	
Sadness	5.343*	1.648*	17.321*	0.005*	4.957*	1.357*	18.107*	0.015*	1.371	0.466	4.039	0.567
Pessimism	1.824	0.817	4.072	0.143	1.733	0.777	3.867	0.179	2.560*	1.028*	6.375*	0.043*
Failure	1.079	0.458	2.543	0.861	1.332	0.560	3.166	0.516	1.696	0.666	4.322	0.268
Lack of pleasure	1.042	0.464	2.338	0.921	0.938	0.419	2.099	0.875	2.000	0.812	4.926	0.132
Guilty feelings	0.840	0.382	1.847	0.665	1.489	0.677	3.273	0.322	3.335*	1.258*	8.838*	0.015*
Punishment feelings	2.222	0.850	5.808	0.103	1.088	0.426	2.783	0.860	2.857*	1.073*	7.606*	0.036*
Self-hatred	1.091	0.455	2.618	0.845	1.616	0.651	4.011	0.301	1.535	0.591	3.988	0.379
Self-criticalness	2.432*	1.087*	5.438*	0.030*	2.284*	1.024*	5.091*	0.043*	1.773	0.716	4.389	0.216
Suicidal thinking	1.667	0.555	5.001	0.362	3.547	0.951	13.228	0.059	2.040	0.660	6.304	0.216
Crying	1.308	0.553	3.093	0.541	2.725*	1.043*	7.119*	0.041*	3.076*	1.202*	7.873*	0.019*
Agitation	1.255	0.570	2.763	0.573	0.614	0.275	1.370	0.234	2.714*	1.025*	7.186*	0.044*
Lack of interest	1.276	0.581	2.802	0.543	0.926	0.423	2.031	0.849	1.167	0.480	2.836	0.734
Indecision	1.182	0.538	2.594	0.677	1.200	0.546	2.639	0.650	1.510	0.619	3.682	0.365
Worthlessness	1.495	0.611	3.656	0.378	1.616	0.651	4.011	0.301	2.631*	1.022*	6.774*	0.045*
Lack of energy	0.573	0.259	1.267	0.169	1.412	0.640	3.115	0.393	1.510	0.619	3.682	0.365
Change in sleep patterns	0.686	0.287	1.643	0.398	2.795*	1.188*	6.575*	0.019*	3.080	0.964	9.839	0.058
Irritability	1.276	0.581	2.802	0.543	1.278	0.581	2.809	0.542	1.433	0.588	3.493	0.428
Appetite changes	1.268	0.578	2.783	0.554	1.190	0.543	2.609	0.664	1.773	0.716	4.389	0.216
Concentration problems	0.491	0.217	1.112	0.088	1.035	0.467	2.297	0.932	0.949	0.386	2.332	0.908
Fatigue	0.852	0.388	1.868	0.689	2.229	0.997	4.984	0.051	1.361	0.559	3.316	0.497
Lack of sex appetite	0.490	0.183	1.312	0.156	0.502	0.197	1.278	0.148	1.428	0.512	3.984	0.497

CI (confidence interval 95 %), * p < 0.05

- Among female study participants, a lower PES was associated with sadness (OR: 5.391, CI 95 %: 1.122–25.903, p < 0.05) self-criticalness (OR: 3.132, CI 95 %: 1.126–8.706, p < 0.05), pessimism (OR: 2.869, CI 95 %: 1.034–7.956, p < 0.05) and feelings of worthlessness (OR: 6.517, CI 95 %: 1.366–31.093, p < 0.05). We found a statistically significant relationship between a lower PCS and pessimism (OR: 3.198, CI 95 %: 1.049–9.751, p < 0.05), guilt feelings (OR: 4.133, CI 95 %: 1.294–13.207, p < 0.05), punishment feelings (OR: 4.300, CI 95 %: 1.359–13.608, p < 0.05) and agitation (OR: 4.230, CI 95 %: 1.098–16.302, p < 0.05). There was a statistically significant relation between loss of friendships in successive waves and sadness (OR: 5.884, CI 95 %: 1.508–22.966, p < 0.05), pessimism (OR: 4.718, CI 95 %: 1.694–13.142, p < 0.05) and self-criticalness (OR: 3.977, CI 95 %: 1.459–10.843, p < 0.05).
- Among male study participants, PES was associated only with changes in sleep pattern (OR: 7.778, CI 95 %: 1.561–38.756, p < 0.05) and PCS with crying (OR: 7.333, CI 95 %: 1.168–46.052, p < 0.05). Loss of friendships had no statistically significant association with any BDI-II symptoms.

In addition, lack of satisfaction with ICS showed some associations with BDI-II symptoms that are not included in Table 3; concretely, loss of energy (OR: 3.022, CI 95 %: 1.350–6.765, p < 0.05) and irritability (OR: 2.329, CI 95 %: 1.057–5.133, p < 0.05).

### Social support and social networks

As expected, we observed that among the social support variables, dissatisfaction with PCS was associated with dissatisfaction with PES and instrumental support, both IES and ICS. In addition, the participants who expressed dissatisfaction with PCS also experienced a loss of friendships over the course of the waves, and at the time of this study, their social networks had fewer than 8 friends according to the MISS. Conflictive relationships with friends were associated with dissatisfaction with PES and with having fewer than 8 friends in one’s social network. Conflictive relations with family members were associated negatively with dissatisfaction with IES.

### Social support preferences by gender

Qualitative analysis revealed that for depressive symptoms, the study participants preferred support from their own social networks, which consisted of two clearly differentiated relational territories: family and peers/friends. Of the 105 participants, only 21 (11 with a diagnosis, 7 with undiagnosed distress, and 3 from the control group) were clearly and explicitly inclined to seek professional help for the symptoms of depression in preference to their own social networks. The study participants felt that their social networks could help to mitigate emotional distress or find ways of resolving it, including by evaluating its severity. There were, however, gender differences with respect to the type and function of social support considered most helpful or preferable for dealing with depressive symptoms. This difference may be summed up as a tendency among the young men in our sample to use their social networks to normalize their emotional distress and thereby bring it under control (“forgetting about it”), while young women study participants tended to understand their social networks as a resource for producing awareness of their emotional distress (“talking about it”). The responses shown in Table 4 support this gendered typology of preferences.

**Table 4:** Preferred social support for symptoms of depression by gender

Preferred social support for young men	Preferred social support for young women
“Don’t talk about what’s bothering [the person]; suggest things to do” 100_ManDiagnosis, BDI-II Minimal (4) “Don’t try to make them talk, just let them know you’re there, without talking about it. [That way] they can put their problems aside for a while and relax” 101_ManDiagnosis, BDI-II Minimal (11) “Go out with friends and laugh. Let’s go out and have a good time, go to the bar, have a beer and forget about everything for a little while, and that’s it. Mutual support, that’s what I think and what my friends want, I think” 103_ManDiagnosis, BDI-II Minimal (10) “Having the support of your friends, having them tell you, Come on, man, wake up, on Friday we’re going here, on Saturday we’re going there. You don’t feel like it? Then we’re coming to your house and we’re staying there” 94_ManDiagnosis, BDI-II Mild (16) “I try to escape with my friends, doing things together, but when I’m in really bad shape I don’t feel like doing anything” 96_ManDiagnosis, BDI-II Severe (32) “I have a friend who, well, I’ve got to thank him, because he’s been there for me. ‘OK, come on, let’s go to the beach. Whatever.’ ‘No, no, I don’t feel like it, I don’t know, no.’ ‘Yes, you’re coming.’ And my mother opens the door and he comes in, and I’m there in bed, [and he says], ‘Come on, you’re coming.’ And we go to the beach and I have a great time. Well, you’re grateful to have friends like that, the ones you can count on the fingers of one hand” 97_ ManDiagnosis, BDI-II Mild (15) “Going out with people, your friends, with somebody, basically, yes. Going to the movies, going out for coffee, meeting up in the afternoon, going to the beach, anything to disconnect a little. Sports help too, I don’t know, anything that helps you disconnect” 114_ManDistress, BDI-II Minimal (7) “Well, I’d go out partying not because I wanted to but because my friends dragged me along, and I don’t think that’s a bad thing. I think I’d do the same thing for them because I think that it’s not about partying but being with other people, forgetting about yourself, and above all having a good time” 118_ManDistress, BDI-II Minimal (5)	“Well, being with me, I mean, supporting me, having people around me who can put themselves in my position and listen to me and understand me, even if they can’t do anything, just being with me [is enough]” 10_WomanDiagnosis, BDI-II Moderate (23) “Girls usually help each other. Sometimes we give each other advice, or we try to get her [the depressed person’s] mind off it, so she won’t think about it. Give her another solution. We talk a lot” 14_WomanDiagnosis, BDI-II Severe (43) “I’d like it to be my friends. If you can see that they’re worried about you, you say, OK, even if I wanted to be by myself I know that I have someone I can talk to, someone I can unburden myself to” 3_WomanDiagnosis, BDI-II Minimal (10) “My friends tell me you can’t go on like that. I don’t know, really, I think that nobody realizes what’s happening to them until they see it. Your friends tell you that you can’t go on like that” 4_WomanDiagnosis, BDI-II Minimal (8) “[I] really appreciated the people who came on their own and said, ‘Hey, what’s going on with you?’ Because there were some people [who said] ‘Oh, I knew you were feeling bad.’ [And when you said] ‘Well, yes, sort of,’ they avoided you. When you’re feeling bad you see who really cares about you or worries about you. That, and talking. Coming over to talk, or saying, ‘Listen, we’re going someplace this weekend, come on’” 5_WomanDiagnosis, BDI-II Minimal (10) “Talking with your friends first, then with your family” 63_WomanDistress, BDI-II Moderate (26) “Friends? They’re pretty important. If you have one kind of problem or another, or if you’re feeling down, what [these situations] always have in common is that you feel alone, right? I think that’s what all these emotional states have in common: loneliness. Your family’s there, but you know they’ll always be there! You know they love you unconditionally, so you need something more that really tells you that you matter as a person, that you have something to give others, that your friends are there for you because they care” 77_WomanDistress, BDI-II Moderate (24) “I spent three years crying until all of a sudden some friends turned up and we started talking about it, and then I went to play basketball with them and little by little things started to change, I started understanding everything, but it was really complicated” 81_WomanDistress, BDI-II Moderate (26)

Words added by the authors to a quotation to improve the reader’s understanding are indicated in square brackets []. Each quotation is followed by a summary of the characteristics of the specific participant, including participant number at the beginning; gender; subgroup; and Beck depression inventory (BDI-II) score

According to our male participants, self-control preceded awareness of the problem. “Forgetting” about their troubles meant going out, having fun, partying. These activities were seen as necessary for taking the edge off emotional distress. As one of the study participants put it, one way to help at such times is “Not to talk about it (the problem), but to suggest things to do.” As a strategy, however, “forgetting about” one’s problems was not entirely incompatible with “talking about” them, but an activity that preceded it. Often “forgetting about” one’s troubles was a necessary first step that made it possible to “talk about” them later, with one’s emotional distress under greater self-control.

By contrast, for the young women in our sample, awareness of the problem preceded self-control. Talking about their problems with friends allowed them to problematize and analyze what was wrong through negotiation with an interlocutor and begin to imagine a solution, preferably one their own social world was capable of generating. In this way, talking about the problem was not understood as a loss of self-control, but as a necessary step for achieving it.

## Discussion

This study shows that among emerging adults depression is associated with dissatisfaction with both psychological everyday support and psychological crisis support, and with the loss of friendships over time. In addition, qualitative analysis demonstrates that the type of social support young people consider most helpful for confronting depressive emotional distress varies by gender and results in different strategies: young men want their social networks to help them achieve self-control, while young women want them to facilitate awareness of their problems.

In this study, we found that dissatisfaction with instrumental support had no statistically significant association with depression, but it was associated with some of its somatic symptoms, such as loss of energy and irritability. Dissatisfaction with psychological support, however, was strongly associated with both depression and with many of its psychological symptoms such as sadness, pessimism, guilt feelings, punishment feelings and self-dislike, among others shown in Table 3.

The connection between depression and lack of social support found in our study is borne out in much of the literature. Several cross-sectional studies have noted that lower levels of perceived social support are associated with higher levels of depressive symptoms in adolescents and young adults [19, 32]. Additionally, several longitudinal studies [33] have demonstrated that lack of social support has predictive value for the appearance of depressive symptomatology. Most studies, however, do not differentiate between perceived psychological support and perceived instrumental support, or their everyday and crisis modalities. Nor do they generally include structural social network variables such as the number of friendships. Non-use of qualitative techniques in research on this subject has limited our knowledge of young people’s perspectives on the most helpful forms of social support in the face of depression.

Our results capture gendered nuances in the kinds of social support young people find most satisfactory for dealing with symptoms of depression and depressive distress. The young men in our sample generally favored a type of peer support that helps them maintain self-control, leaving to one side awareness of the problem and, as a consequence, reflection on it. This may help to explain why young men in general have a lower level of mental health literacy [34], and why they are more likely to avoid using professional mental health services in the course of help-seeking, a tendency widely reported in the literature [7–10, 34]. Efforts to maintain self-control may also be related to higher consumption of psychoactive substances, as the self-medication hypothesis suggests [35]. In an analysis of the same sample, we observed that tobacco consumption was associated with depressive and anxiety symptoms among young men, but not among young women, and that in general the smokers with depressive symptoms explained that they used tobacco as a form of self-medication [36]. By contrast, the preferred strategy among the young women in our sample was to mobilize their social networks, especially peers, to talk about what was troubling them as a way of bringing the sources of their distress to conscious awareness and problematizing their symptoms. A possible explanation for this preference is the greater association we found among the female study participants between dissatisfaction with psychological support and a range of symptoms, especially of a psychological nature.

As some studies have pointed out [37] depression usually develops in the context of two kinds of situations: lack of personal autonomy, and loss of social support. One may balance out the other; a gain in personal autonomy may reduce the need for social support, while a gain in social support may compensate for a loss of personal autonomy. Some studies, however, have shown that the role played by autonomy and social support is mediated to a great extent by culture and cultural models of gender. For example, a recent comparative study [38] of German and Turkish women found that satisfaction with social support predicted better mental health in Turkish women, while among German women autonomy was a better mental health predictor.

One of the limitations of this study with respect to its quantitative results is clearly the size of our sample. In qualitative research, however, small sample size is usual, and ours is well within normal range. Another limitation is the primarily cross-sectional nature of the study, although we included some longitudinal variables such as the loss of friendships through time. For this reason, for example, it is possible that depression itself may influence perceived social support, or vice versa. A further limitation is the use of different instruments; in waves 1 and 4, depression was established by a medical diagnosis, but the BDI-II was not administered. For this reason it was not possible to analyze the development of depressive symptoms through time using the same standardized instrument. An additional limitation is that, in our analysis, we did not adjust for the number of tests used in order to correct the type 1 error rate inflation, for instance Bonferroni correction, which adjusts for inflation of the family-wise error rate by dividing the alpha value −0.05—by the number of tests used. For this reason the results need to be interpreted with caution. Finally, our study shares a limitation with the majority of studies of social support, defining this concept as perceived social support. Because of the difficulty involved in analyzing this psychosocial factor, studies generally do not include data derived from observation, or data on social support actually received that can be triangulated with perceived social support. In this study, however, we have tried to compensate for the absence of this type of data by using social network structural variables, distinguishing between psychological and instrumental support, and data derived from qualitative analysis. Despite the limitations we have identified, we believe that the integrated methodology of this study produces results that may be useful for future studies of depression and social ties in adolescents and emerging adults.

To the best of our knowledge, no other studies of adolescents or emerging adults have used qualitative research methods to explore the kind of social support young people consider most helpful in resolving depressive symptoms. Our study thus fills a void in mental health knowledge through the use of mixed (quantitative/qualitative) methods to investigate the relationship between social ties and depression.

Depression is the fourth leading disorder worldwide in terms of disease burden, and it is projected that by the year 2030 it will probably be the first [39, 40]. Depression in adolescents and emerging adults is an even more serious health problem because of its tendency to persist into adulthood in the form of severe mental disorder. This study contributes to our knowledge of the impact of social support on depression and depressive symptoms in this age group, and shows that mental health interventions should take gender differences into account.

## Conclusions

This exploratory study yielded clear results of two types regarding the complex relation between social ties and depression among adolescents and emerging adults. First, loss of friendships over time and dissatisfaction with social and psychological support are variables associated with depression in this age group. Second, there are gender differences both in strategies for managing distress, and in how social support was understood to mitigate depressive symptoms. Male study participants prioritized support that helped them achieve self-control as a first step toward awareness of their emotional distress, while female study participants prioritized support that helped them achieve awareness of the problem as a first step toward self-control. Treatment for emerging adults with depression should take into account not only the impact of social support, but also gender differences in what they consider to be the most appropriate form of social support for dealing with emotional distress.

## Additional file

10.1186/s13034-015-0088-x The qualitative questionnaire.
